# *Lactuca capensis* reverses memory deficits in Aβ1‐42‐induced an animal model of Alzheimer's disease

**DOI:** 10.1111/jcmm.13299

**Published:** 2017-08-16

**Authors:** Paula Alexandra Postu, Jaures A. K. Noumedem, Oana Cioanca, Monica Hancianu, Marius Mihasan, Mitica Ciorpac, Dragos Lucian Gorgan, Brindusa Alina Petre, Lucian Hritcu

**Affiliations:** ^1^ Department of Biology Alexandru Ioan Cuza University of Iasi Iasi Romania; ^2^ Pôle Recherche Innovation et Entrepreneuriat (PRIE) Institut Universitaire de la Côte Douala Cameroon; ^3^ Faculty of Pharmacy University of Medicine and Pharmacy ‘Gr. T. Popa’ Iasi Romania; ^4^ Department of Chemistry Alexandru Ioan Cuza University of Iasi Iasi Romania

**Keywords:** *Lactuca capensis* extract, amyloid, antioxidant, brain‐derived neurotrophic factor, IL‐1β, dementia

## Abstract

We investigated the neuropharmacological effects of the methanolic extract from *Lactuca capensis* Thunb. leaves (100 and 200 mg/kg) for 21 days on memory impairment in an Alzheimer's disease (AD) rat model produced by direct intraventricular delivery of amyloid‐β1‐42 (Aβ1‐42). Behavioural assays such as Y‐maze and radial arm maze test were used for assessing memory performance. Aβ1‐42 decreased cognitive performance in the behavioural tests which were ameliorated by pre‐treatment with the methanolic extract. Acetylcholinesterase activity and oxidant–antioxidant balance in the rat hippocampus were abnormally altered by Aβ1‐42 treatment while these deficits were recovered by pre‐treatment with the methanolic extract. In addition, rats were given Aβ1‐42 exhibited in the hippocampus decreased brain‐derived neurotrophic factor (BDNF) mRNA copy number and increased IL‐1β mRNA copy number which was reversed by the methanolic extract administration. These findings suggest that the methanolic extract could be a potent neuropharmacological agent against dementia via modulating cholinergic activity, increasing of BDNF levels and promoting antioxidant action in the rat hippocampus.

## Introduction

Alzheimer's disease (AD) is an irreversible brain disorder mainly characterized by cholinergic deficits, amyloid/tau toxicity and oxidative stress/mitochondrial dysfunctions 1. Various peptide fragments amyloid beta 1‐42 (Aβ1‐42) resulted from the abnormal cleavage of amyloid precursor protein (APP) generate deposits in the specific area of the brain leading to losing of memory and cognitive impairment in patients with AD 2. In addition, the peripheral anionic site (PAS), located on the acetylcholinesterase (AChE) enzyme, was previously reported as a facilitator factor that induced aggregation of the Aβ‐monomers 3. The Aβ‐AChE complexes were found to enhance neurodegenerative changes induced by Aβ peptide related to AD neuropathology 4. AChE is a target for AD therapy and as inhibiting of its activity helps to maintain the acetylcholine (ACh) levels in the neuronal synapses with positive effects in patients with AD 5. Evidence for Aβ1‐42‐induced oxidative stress suggested high levels of oxygen species, lipid peroxidation, protein oxidation, DNA and RNA damage 2, 6. Moreover, we have previously demonstrated a relationship between memory loss, anxiety, depression, oxidative stress and Aβ1‐42 using a rat model of AD 7, 8, 9.

Brain‐derived neurotrophic factor (BDNF) sustains the neurons viability and their connections that are vulnerable to diseases of the ageing brain such as AD 10. There is a strong evidence that a down‐regulation of BDNF mRNA and protein related to cognitive decline has been demonstrated in AD 10, 11. It has been documented that Aβ reduces BDNF/TrkB receptor levels and decreases TrkB‐mediating signalling 12. A deficit of BDNF in the hippocampus has been shown in both the animal model and patients with AD 13. Additionally, neuroinflammation plays an important role starting with the early stages of AD that precede dementia stage 14. Evidence suggested that astrocytes and produce microglia cytokines‐induced neuroinflammation in AD. In ageing TgAPPsw and PSAPP transgenic mice, accumulation of Aβ is correlated with increased levels of proinflammatory cytokines such as TNF‐α, interleukin‐6, interleukin‐1α, GM‐CSF 15. Moreover, exposure of microglia to pre‐aggregated Aβ1‐42 peptide induces increased levels of proinflammatory cytokines such as pro‐interleukin‐1β, interleukin‐6 and TNF‐α 16.

Among numerous herbal plants known to have medicinal value is *Lactuca capensis* Thunb., widespread in tropical Africa, Madagascar, South Africa and Yemen. *Lactuca* L. genus belongs to the family Asteraceae and comprises ~100 wild species 17. In African countries, *Lactuca capensis* is traditionally used as vegetable and herbal remedy 17. It has been reported that the leaves of *Lactuca capensis* are lightly boiled and eaten in Zimbabwe, while in Lesotho is used as a potherb as well as in Madagascar. Venereal diseases are cured using an infusion of the leaves in northern Nigeria or of the roots in Kenya. For treating sores, ulcers, leprosy and eczema are used pulverized roots in Congo 18. *Lactuca sativa* is used in folk medicine in the treatment of anxiety, insomnia, neurosis, dry coughs and rheumatic pain 19. Sayyah *et al*. 20 reported that *Lactuca sativa* seed extract exhibited analgesic and anti‐inflammatory activity in laboratory rats. The authors attributed these effects to simple phenols and saponins compounds demonstrated in several plant extracts. The anxiolytic properties of the hydro‐alcoholic extract of the *Lactuca sativa* leaves in mice were reported by Harsha and Anilakumar 21. *Lactuca sativa* acts as an antioxidant in preventing either cancer‐ 22 and D‐galactose‐induced oxidative stress 23 in mice.

Recently, Michalska and Kisiel 17 reported the presence of the coumarins scopolin, isofraxoside and α‐xylofuranosyluracil in the roots and aerial parts of the *Lactuca capensis*. Scopolin was found to sustain memory formation, acetylcholine releasing from brain synaptosomes and induce long‐term potentiation in the rat hippocampus 24. Also, scopolin is an inhibitor of the acetylcholinesterase activity, being a good candidate for AD therapy 25. Despite the many uses of *Lactuca* species, to date, there is no scientific paper clarifying the after‐effects of the methanolic extract from *Lactuca capensis* leaves on memory processes and oxidative level in the rat hippocampus of the Aβ1‐42‐induced an AD rat model. Herein, we investigated several neuropharmacological consequences of the methanolic extract from *Lactuca capensis* leaves using Aβ1‐42‐induced cognitive deficits associated with brain oxidative damage in laboratory rats.

## Materials and methods

### Plant material and plant extract

*Lactuca capensis* leaves were collected in Dschang, West Region of Cameroon, in June 2010 and identified by Mr. Victor Nana at the National Herbarium Yaoundé where a voucher specimen (no. 27743 HNC) was registered and stored for ready reference. *Lactuca capensis* leaves were normally dried in the air and pulverized into a fine powder. One thousand grams of pulverized sample material was macerated in methanol for 48 hrs at room temperature (25°C), and then the mixture was filtered through Whatman filter paper no. 1. The crude extract was first lyophilized and then dissolved in distilled water and administered by gastric gavage to animals in two different doses, 100 and 200 mg/kg body weight.

### HPLC analysis

HPLC analysis of the methanolic extract from *L. capensis* leaves was performed using a Thermo UltiMate3000 (Thermo Fisher Scientific, USA) gradient chromatograph equipped with quaternary pumps controlled by Chromeleon interface, an autosampler and multidiode array detector (DAD). Solvents were filtered using a Millipore system and analysis was performed on an Accucore XL C18 column (150 × 4.6 mm, 4 μm). All the samples were filtered through 0.22 μm filter before being analysed. The mobile phase was pure acetonitrile (A) and bidistilled water containing 0.1% acetic acid (B), and the gradient setting was 10–23% (A) in 5 min.; 23% (A) isocratic for 10 min. and then 23–35% (A) in 12 min.; 35–70% (A) for 5 min. The injection volume was 20 μl with scanning absorbance wavelengths from 240 to 520 nm, typical for phenols including flavonols, flavones, hydroxycinnamic acids and anthocyanins. The flow rate increased from 0.2 ml to 1 ml/min. HPLC grade solvents and bidistilled water were used in the chromatographic studies. All chromatographic experiments were performed at 25°C. Standard curves for authentic samples of the polyphenols were obtained from purchased reagents (Sigma Chemical Co., St. Louis, USA) of analytical or high‐performance liquid chromatography (HPLC) grade. Each solution was injected in triplicate and the calibration curves were constructed with the averages.

### Animals

We have started the experiment using twenty 4‐ to 5‐month‐old male Wistar rats weighing 350 ± 10 g. The animals were housed at an appropriate temperature and light‐controlled room (22°C, a 12‐hrs cycle starting at 08:00 hrs) and were fed and allowed to drink water *ad libitum*. The experiments were conducted in the quiet laboratory between hours of 09:00 to 15:00. The rats were divided into four groups (five animals per group): (1) the control group (sham‐operated) received the distilled water treatment; (2) the Aβ(1‐42)‐alone‐treated group received the distilled water treatment, as negative control; (3) the Aβ(1‐42)‐treated group received 100 mg/kg of the methanolic extract from *L. capensis* leaves treatment [Aβ(1‐42)+L100] and (4) the Aβ(1‐42)‐treated group received 200 mg/kg of the methanolic extract from *L. capensis* leaves treatment [Aβ(1‐42)+L200]. The methanolic extract from *L. capensis* leaves was dissolved in distilled water. The administration of the distilled water and the methanolic extract was performed by 15‐gauge oral gavage needle (Instech, Plymouth Meeting, PA, USA). The volume administered was 10 ml/kg of body weight, daily, for 21 consecutive days after neurosurgery. Moreover, animals received additional extract treatment during entire training in the Y‐maze and the radial arm maze tasks. The methanolic extract doses (100 and 200 mg/kg) used in this experiment were chosen as they have been demonstrated by our group to provide significant effects on memory formation in an amyloid beta (1‐42)‐induced Alzheimer's disease rat model 26. Rats were treated in accordance with the guidelines of animal bioethics from the Act on Animal Experimentation and Animal Health and Welfare from Romania, and all procedures were in compliance with Directive 2010/63/EU of the European Parliament and of the Council of 22 September 2010 on the protection of animals used for scientific purposes. This study was approved by the Committee on the Ethics of Animal Experiments of the Alexandru Ioan Cuza University of Iasi, Faculty of Biology (Permit Number: 2196) and also, efforts were made to minimize animal suffering and to reduce the number of animals used.

### Neurosurgery

For stereotaxic surgery, rats were anaesthetized using sodium pentobarbital (50 mg/kg b.w., i.p., Sigma‐Aldrich, Schnelldorf, Germany) and then were mounted in the stereotaxic apparatus with the nose oriented 11° below horizontal zero plane. The scalp was cleaned with iodine solution and incised on the midline, and a burr hole was drilled through the skull. An animal model of AD was established by intracerebroventricular (i.c.v.) injection of beta‐amyloid peptide [Aβ(1‐42), Rat, Sigma‐Aldrich] solution. Aβ(1‐42) peptide was dissolved in sterile physiological saline solution (1 mM) in the tube, which was then sealed and incubated at 37°C for 4 days to cause the peptide to aggregate. Aβ(1‐42) was administered right‐unilaterally through a Hamilton syringe over 4 min., and the syringe was left in place for 5 min. after injection before being slowly removed. The injection volume (4 μl) was delivered gradually (1 μl/min.) using the following coordinates: 1.5 mm lateral to the midline; 7.4 mm ventral to the surface of the cortex, according to previous literature 27. The sham‐operated rats received 4 μl of 0.9% sterile physiological saline solution instead of Aβ(1‐42) peptide solution. Postoperatively, the rats were given special care until spontaneous feeding was restored. Behavioural tests were conducted 2 weeks after neurosurgery and were performed blind to the treatments by the observer.

### Y‐maze task

According to previous protocols, spatial recognition memory was assessed by recording spontaneous alternation behaviour in a single‐session Y‐maze on the 14th day post‐surgery 28, 29. The Y‐maze used in this study was made of black Plexiglas and consisted of three arms (35 cm long, 25 cm high and 10 cm wide) and an equilateral triangular central area. 30 min. after the administration of methanolic extract, rats were placed at the end of one arm and allowed to move freely through the maze for 8 min. The series of arm entries were recorded visually. An arm entry was counted when the hind paws of the rat were completely within the arm. Spontaneous alternation behaviour was defined as entry into all three arms on consecutive choices. The number of maximum spontaneous alternation behaviours was then the total number of arms entered −2 and per cent spontaneous alternation was calculated as (actual alternations/maximum alternations) × 100. Locomotor activity was assessed by the number of arm entries. The maze was cleaned with a 10% ethanol solution between animal trials.

### Radial arm maze task

Spatial memory was tested using a radial arm maze during 7‐day period starting with 16th day post‐surgery 29, 30. The radial arm maze used here consisted of eight arms, numbered from 1 to 8 (48 cm × 12 cm), with an extending radially from the central area of 32 cm in diameter. The system was placed 50 cm above the floor and surrounded by several extra‐maze visual cues placed at the same position during the test. At the end of each arm, there was a food cup containing a single 50‐mg food pellet. Prior to the performance of the maze task, the animals were kept on restricted diet and body weight was maintained at 85% of their free‐feeding weight over a week period, with water being available *ad libitum*. Before the actual training began, three or four rats were simultaneously placed in the radial arm maze and allowed to explore for 5 min. and take the food freely. The food was initially available throughout the maze but was gradually restricted to the food cup. The animals were trained for 4 days to run to the end of the arms and consume the bait. To evaluate the basal activity of rats in radial arm maze, the rats were given five consecutive training trials per day to run to the end of the arms and consume the bait. The training trial continued until all five baits have been consumed or until the 5 min. has elapsed which represent important performance criteria. After adaptation, all rats were trained with only one trial per day. Briefly, 30 min. after the administration of the methanolic extract, each animal was placed individually in the centre of the maze and subjected to working and reference memory tasks, in which same five arms (nos. 1, 2, 4, 5 and 7), were baited for each daily training trial. The other three arms (nos. 3, 6 and 8) were never baited. The selection of the baited arms is based on the fact that animals prefer to solve the maze using an immediate adjacent arm selection strategy. In this case, we altered adjacent arm patterning behaviour by only baiting five arms (nos. 1, 2, 4, 5 and 7) subjecting animals to change their strategy and avoid the unbaited arms. An arm entry was counted when all four limbs of the rat were within an arm. The series of arm entries were recorded visually. Measures were made by (1) counting the number of working memory errors (entering an arm containing food, but previously entered) and (2) evaluating the reference memory errors by counting animal enters in an arm without bait. Reference memory is view as a long‐term memory for information that remains constant over repeated trials (memory for the positions of baited arms), whereas working memory is considered a short‐term memory in which the information to be remembered changes in every trial (memory for the positions of arms that had already been visited in each trial). The maze was cleaned each time with a 10% ethanol solution and dried with a cloth before the next animal was tested.

### Biochemical parameter assay

After behavioural tests, all rats were deeply anaesthetized (using sodium pentobarbital, 100 mg/kg b.w., i.p., Sigma‐Aldrich) and decapitated and whole brains were removed. The hippocampi were precisely excised. The hippocampal samples were weighed and individual homogenized (1:10) using a Potter Homogenizer coupled with Cole‐Parmer Servodyne Mixer in ice‐cold 0.1 M potassium phosphate buffer (pH 7.4), containing 1.15% KCl. The homogenate was centrifuged (15 min. at 960× g), and the supernatant was used for testing AChE‐, SOD‐ and GPX‐specific activities, the total content of reduced GSH, MDA and protein carbonyl levels.

### Determination of hippocampal AChE activity

The activity of acetylcholinesterase (AChE) in the rat hippocampus was determined according to the previously described method by Ellman *et al*. 31 using acetylthiocholine (ATC) as artificial substrate 32. The reaction mixture (600 μl final volume) contained 0.26 M phosphate buffer with pH 7.4, 1 mM 5,5′‐dithiobis‐2‐nitrobenzoic acid (DTNB) and 5 mM ATC chloride. The assay was started by adding the supernatant and following the appearance of a yellow colour at 412 nm for 10 min. at room temperature. Suitable controls were performed for the non‐enzymatic hydrolysis of ATC. The enzyme activity was expressed as nmol of ACT/min. per/mg of protein.

### Determination of hippocampal SOD activity

The activity of superoxide dismutase (SOD, EC 1.15.1.1) was assayed by monitoring its ability to inhibit the photochemical reduction of nitroblue tetrazolium (NBT). 200 μl of supernatant was added to 1.5 ml reaction mixture containing 100 mM TRIS/HCl (pH 7.8), 75 mM NBT, 2 μM riboflavin and 6 mM EDTA. The production of blue formazan was monitored by an increase in absorbance at 560 nm. As previously described by Winterbourn *et al*. 33, one unit of SOD was defined as the quantity required to inhibit the rate of NBT reduction by 50%. The enzyme activity was expressed as units/mg protein.

### Determination of hippocampal GPX activity

Glutathione peroxidase (GPX, E.C. 1.11.1.9) activity was analysed by a spectrophotometric assay. A reaction mixture consisting of 1 ml of 0.4 M phosphate buffer (pH 7.0) containing 0.4 mM EDTA, 1 ml of 5 mM NaN_3_, 1 ml of 4 mM glutathione (GSH) and 200 μl of supernatant was pre‐incubated at 37°C for 5 min. Then, 1 ml of 4 mM H_2_O_2_ was added and incubated at 37°C for further 5 min., as previously described by Sharma and Gupta 34, the excess amount of GSH was quantified using the 5,5′‐dithiobis‐2‐nitrobenzoic acid (DTNB). One unit of GPX was defined as the amount of enzyme required to oxidize 1 nmol GSH/min. The enzyme activity was expressed as units/mg protein.

### Total hippocampal content of reduced GSH

Glutathione (GSH) was measured following the method of Fukuzawa and Tokumura 35. 200 μl of supernatant was added to 1.1 ml of 0.25 M sodium phosphate buffer (pH 7.4) followed by the addition of 130 μl DTNB 0.04%. Finally, the mixture was brought to a final volume of 1.5 ml with distilled water and absorbance was read at 412 nm using a spectrophotometer. The results were expressed as μg GSH/μg protein.

### Determination of hippocampal protein carbonyl level

The extent of protein oxidation in the hippocampus was assessed by measuring the content of protein carbonyl groups, using 2,4‐dinitrophenylhydrazine (DNPH) derivatization as described by Oliver *et al*. 36 and modified by Luo and Wehr 37. Basically, the supernatant fraction was divided into two equal aliquots containing both, ~2 mg of total protein. Both aliquots were precipitated using 10% trichloroacetic acid (TCA, w/v, final concentration). One sample was treated with 2 N HCl, and the another sample was treated with an equal volume of 0.2% (w/v) DNPH in 2 N HCl. Both samples were incubated at 25°C and stirred at 5‐min. intervals. The samples were then reprecipitated with 10% TCA (final concentration) and subsequently extracted with ethanol‐ethyl acetate (1:1, v/v) and then reprecipitated at 10% TCA. The pellets were carefully drained and dissolved in 8 M urea with 20 mM sodium phosphate buffer, pH 6.5. Insoluble debris was removed by centrifugation at 13,000× g at 4°C. The absorbance at 370 nm of the DNPH‐treated sample versus the HCl control was recorded, and the results were expressed as nmols of DNPH incorporated/mg of total protein based on an average absorptivity of 21 per mM per cm for most aliphatic hydrazones.

### Determination of hippocampal MDA level

Malondialdehyde (MDA), which is an indicator of lipid peroxidation, was spectrophotometrically measured using the thiobarbituric acid assay as previously described by Ohkawa *et al*. 38. 200 μl of supernatant was added and briefly mixed with 1 ml of 50% trichloroacetic acid in 0.1 M HCl and 1 ml of 26 mM thiobarbituric acid. After vortex mixing, samples were maintained at 95°C for 20 min. Afterwards samples were centrifuged at 960× g for 10 min. and supernatants were read at 532 nm. A calibration curve was obtained using MDA as standard, and the results were expressed as nmol/mg protein.

### Estimation of protein concentration

Estimation of protein was performed using a bicinchoninic acid (BCA) protein assay kit from Sigma‐Aldrich. The BCA protein assay is a detergent‐compatible formulation based on BCA for the colorimetric detection and quantification of total protein, as previously described by Smith *et al*. 39.

### Determination of DNA fragmentation (apoptosis)

Here, the determination of histone‐associated DNA fragments was performed using a Cell Death Detection ELISA kit (Roche Diagnostics, Mannheim, Germany) as an indicator of apoptosis according to the protocol sheet and a procedure previously described by Afshin‐Majd *et al*. 40. The assay is based on quantitative sandwich‐enzyme‐immunoassay principle using mouse monoclonal antibodies directed against DNA and histone, respectively. This allows the specific determination of mono‐ and oligonucleosome (histone‐associated DNA fragments) in the fraction of tissue lysates. A number of nucleosomes demonstrating DNA fragmentation were quantified by peroxidase (POD) retained in the immunocomplex. POD was determined photometrically at 405 nm using 2,2′‐azino‐bis (3‐ethylbenzothiazoline‐6‐sulphonic acid) as a substrate by a microplate reader (BioTek, Winooski, Vermont, USA) after 15 min. of substrate reaction time. The enrichment factor was calculated according to the manufacturer's instructions. Enrichment factor = (absorbance of the sample/absorbance of negative control).

### RNA isolation and hippocampal real‐time quantitative PCR (qRT‐PCR)

Twenty seven to thirty milligrams frozen hippocampal tissues from twenty rats were homogenated with 175 μl of RNA Lysis buffer using a ball mill (Mikro‐Dismembrator U; Sartorius, New York, USA) for fine grinding. Total RNA was isolated and purified using SV Total RNA Isolation System kit (Promega, Madison, USA) according to the manufacturer instructions. RNA concentration and integrity were determined by spectrophotometry, checking the A260/A280 ratio to assess the purity of RNA. All samples exhibit an A260/A280 ratio ≥1.8, having no intra‐ or intergroup differences. The reverse transcription and real‐time amplification were performed in single‐step amplification reactions using GoTaq^®^ 1‐Step RT‐qPCR System (Promega) on a 5‐plex HRM Rotor‐Gene 6000 (Corbett, California, USA) rotary real‐time PCR. The reaction was carried out in a 20 μl total volume containing GoTaq^®^ Probe qPCR Master Mix 2X (Promega), GoScript^™^ RT Mix for 1‐Step RT‐qPCR 50X, forward and reverse primers, 100 ng of tRNA template and nuclease‐free water up to volume. To ensure that the amplification reaction was free of contaminants a ‘no template control’ was included for each amplified gene. To increase the qRT‐PCR accuracy, two independent runs were made with three replicates for each individual RNA sample.

The absolute expression level of two genes, BDNF and IL‐1β (Interleukin‐1 beta) was assessed. Pre‐designed specific primers for *Rattus norvegicus* were used as following: BDNF exon 5—300 nM forward and reverse primer (F: 5′‐ATT ACC TGG ATG CCG CAA AC‐3′; R: 5′‐TGA CCC ACT CGC TAA TAC TGT‐3′, 101‐bp product size); IL‐1β—300 nM forward and reverse primer (F: 5′‐AGC ACC TTC TTT TCC TTC ATC TT‐3′, R: 5′‐CAG ACA GCA GGC ATT TT‐3′, 144‐bp product size). The thermal profile was reverse transcription step—15 min. at 37°C; RT inactivation/Hot‐Start activation—10 min. at 95°C; 40 cycles of three‐step qPCR—10 sec. at 95°C for denaturation, 30 sec. at 60°C for align and data collection (Green channel—SYBR Green 1 dye), 30 sec. at 72°C for elongation; and dissociation step from 60 to 95°C.

Via PCR method with the above primers, we obtained the cDNA standards for both amplified genes. The PCR products integrity was checked by agarose gel electrophoresis, purified using Agencourt AMPure XP (Beckman Coulter, Brea, USA) and quantify by nano‐drop spectrophotometry. Standard curves were performed in duplicates, in the same run with samples. Each standard curve had six 10‐fold serial dilutions in duplicates starting at 9.64 × 106 copies of BDNF and, respectively, 6.54 × 106 copies of IL‐1β. Both qRT‐PCR runs exhibit a reaction efficiency between 90% and 100% and *R* ^2^ values higher than 0.98 for standard curves. The absolute expression level of each assayed gene was calculated per experimental versions (group treatments) as a concentration mean (copies/reaction) of twelve expression values (two brain samples per group with two RNA extractions and qRT‐PCR three replicates each).

Levels of BDNF and IL‐1β were determined by absolute quantification using Rotor‐Gene Q‐Pure Detection Software v. 2.2.3. (Qiagen, California USA).

### Statistical analysis

The animal's behavioural activities in the Y‐maze and the radial arm maze tasks and the results for biochemical parameter assay, DNA fragmentation and qRT‐PCR were statistically analysed by two‐way analysis of variance (anova) followed by Tukey's *post hoc* test using GraphPad Prism 6 software for Windows, La Jolla, CA, USA. To evaluate differences between groups in the radial arm maze task, separate repeated‐measures anova were calculated on the number of working memory errors and the number of reference memory errors with group [control, Aβ(1‐42), Aβ(1‐42)+L100 and Aβ(1‐42)+L200] as between‐subject factor and days (1–7) as within‐subjects factors. All results are expressed as a mean ± standard error of the mean (S.E.M). *F* values for which *P* < 0.05 were regarded as statistically significant. Pearson's correlation coefficient and regression analysis were used to evaluate the connection between behavioural measures, the antioxidant defence, BDNF mRNA copy number and lipid peroxidation.

## Results

### Chemical composition of the methanolic extract from *L. capensis* leaves

**Figure 1:**
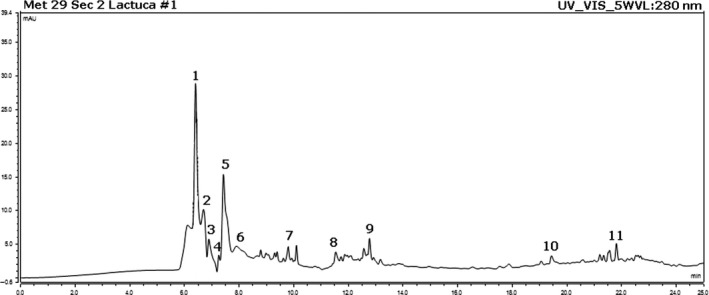
Representative HPLC‐DAD chromatography profile at 280 nm for the flavonoids and hydroxycinnamic acids of the methanolic extract from *Lactuca capensis* leaves: (1) catechin (peak 1); (2) rutoside (peak 2); (3) caffeic acid (peak 3); (4) cyanidol (peak 4); (5) rosmarinic acid (peak 5); (6) cinnamic acid (peak 6); (7) quercetin‐3‐arabinoside (peak 7); (8) luteolin (peak 8); (9) quercetin (peak 9); (10) apigenin (peak 10) and (11) kaempferol (peak 11).

The HPLC results indicated the presence of several flavonoids and hydroxycinnamic acids. Moreover, catechin and cyanidol were also identified in the sample (Fig. 1).

The calculated quantities (mg/dry weight) were slightly different from other literature data 17, but this is not uncommon for lettuce varieties of a particular provenience where soil properties, fertilization, irrigation, light, postharvest handling, and storage influence secondary metabolites quality and quantity. Our data showed similarity in regard to the fact that phenolic acid fractions were dominant in the lettuce leaves as compared to flavonoids. However, higher quantities of catechins were also present in our sample (Table 1). Such compounds may, at least in part, explain the biologic activity of our extract.

**Table 1:** Compounds identified in the methanolic extract from *Lactuca capensis* leaves

Compound	Concentration (mg/g dry extract)
Catechin (1)	2.4958
Rutoside (2)	2.5915
Caffeic acid (3)	1.7579
Cyanidol (4)	0.1472
Rosmarinic acid (5)	5.2784
Cinnamic acid (6)	0.1627
Quercetin‐3‐arabinoside (7)	0.7535
Luteolin (8)	0.2088
Quercetin (9)	0.0162
Apigenin (10)	0.6121
Kaempferol (11)	0.0135

### Spontaneous alternation in Y‐maze task

**Figure 2:**
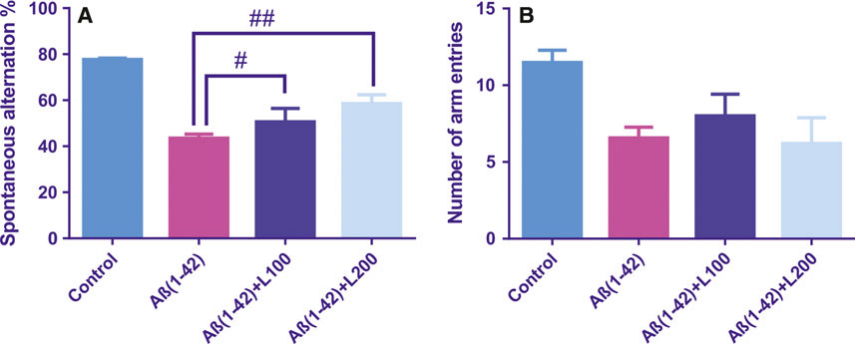
Effects of the methanolic extract from *Lactuca capensis* leaves (100 and 200 mg/kg) administration in the Y‐maze on the spontaneous alternation % (**A**) and the number of arm entries (**B**) in the Aβ1‐42‐treated rats. Values are means ± S.E.M. (*n* = 5 animals per group). For Tukey's *post hoc* analyses— ^#^Aβ1‐42 *versus* Aβ1‐42+L100: *P* < 0.01 and ^##^Aβ1‐42 *versus* Aβ1‐42+L200: *P* < 0.01 (**A**).

The performance of rats in the Y‐maze task was studied to determine spatial recognition memory and is presented in the Figure 2. Significant overall differences between all rat groups [*F*(3, 16) = 16.72, *P* < 0.0001] were observed by analysing the spontaneous alternation percentage within Y‐maze task (Fig. 2A). The spontaneous alternation percentage was found to be significantly decreased for Aβ(1‐42) group (*P* < 0.0001) as compared to the sham‐operated control group. Moreover, both doses of the methanolic extract, but especially 200 mg/kg, increased significantly the spontaneous alternation percentage for *L. capensis*‐pre‐treated Aβ(1‐42) groups, as compared to the Aβ(1‐42) group. To circumvent a compounding effect on locomotor activity on memory performance of rats in the Y‐maze test, the total number of entries was used as an indicator of locomotor activity. No significant differences among *L. capensis*‐pre‐treated Aβ(1‐42) groups and Aβ(1‐42) group were observed (Fig. 2B).

### Spatial memory in radial arm maze task

**Figure 3:**
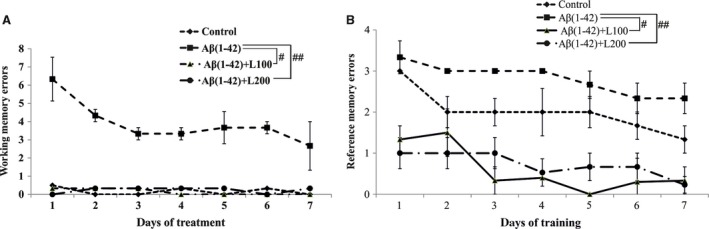
Effects of the methanolic extract from *Lactuca capensis* leaves (100 and 200 mg/kg) administration on the working memory errors (**A**) and the reference memory errors (**B**) during 7‐day training in the radial arm maze in the Aβ1‐42‐treated rats. Values are means ± S.E.M. (*n* = 5 animals per group). For Tukey's *post hoc* analyses—^#^Aβ1‐42 *versus* Aβ1‐42+L100: *P* < 0.0001 and ^##^Aβ1‐42 *versus* Aβ1‐42+L200: *P* < 0.0001 (**A**) and ^#^Aβ1‐42 *versus* Aβ1‐42+L100: *P* < 0.0001 and ^##^Aβ1‐42 *versus* Aβ1‐42+L200: *P* < 0.0001 (**B**).

Analyses of working memory errors within radial arm maze task showed important overall differences between all groups [*F*(3, 16) = 10.82, *P* < 0.0001; Fig. 3A]. Additionally, repeated‐measures anova revealed a significant time difference [*F*(6, 56) = 70.80, *P* < 0.0001], a significant group difference [*F*(3, 56) = 10.89, *P* < 0.0001] and a significant time–group interaction [*F*(18, 56) = 16.37, *P* < 0.0001] for working memory errors evaluation.

Analyses of reference memory errors within radial arm maze task showed significant overall differences between all groups [*F*(3, 16) = 16.42, *P* < 0.0001; Fig. 3B]. Additionally, repeated‐measures anova revealed a significant time difference [*F*(6, 56) = 27.34, *P* < 0.01] and a significant group difference [*F*(3, 56) = 13.25, *P* < 0.0001] for reference memory errors.

### Effects of the methanolic extract from *L. capensis* leaves on cholinesterase inhibitory activity

**Figure 4:**
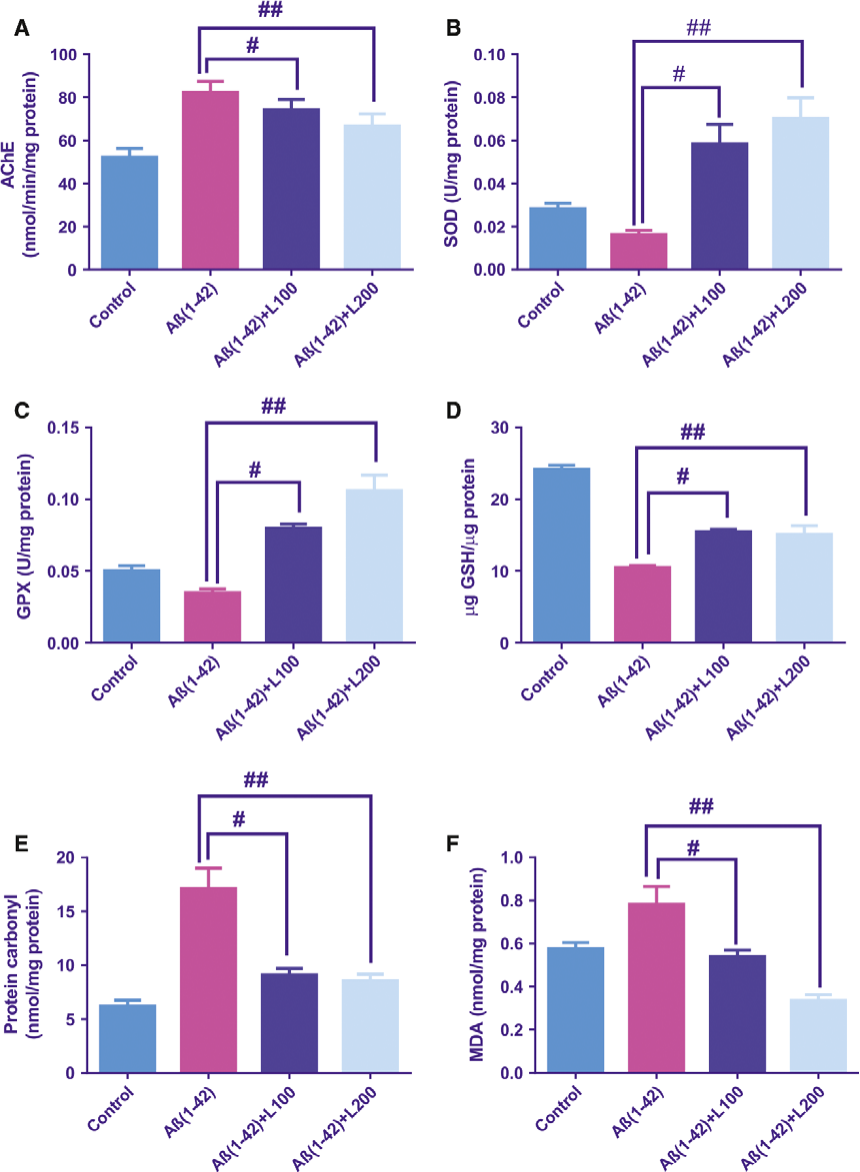
Effects of the methanolic extract from *Lactuca capensis* leaves (100 and 200 mg/kg) administration on the AChE (**A**), SOD‐specific (**B**) and GPX (**C**)‐specific activities, the total content of reduced GSH (**D**), protein carbonyl (**E**) and MDA (**F**) levels estimated in the rat hippocampal homogenates of the Aβ1‐42‐treated rats. Values are means ± S.E.M. (*n* = 5 animals per group). For Tukey's *post hoc* analyses—^#^Aβ1‐42 *versus* Aβ1‐42+L100: *P* < 0.01 and ^##^Aβ1‐42 *versus* Aβ1‐42+L200: *P* < 0.01 (**A**), ^#^Aβ1‐42 *versus* Aβ1‐42+L100: *P* < 0.01 and ^##^Aβ1‐42 *versus* Aβ1‐42+L200: *P* < 0.001 (**B**), ^#^Aβ1‐42 *versus* Aβ1‐42+L100: *P* < 0.001 and ^##^Aβ1‐42 *versus* Aβ1‐42+L200: *P* < 0.0001 (**C**), ^#^Aβ1‐42 *versus* Aβ1‐42+L100: *P* < 0.001 and ^##^Aβ1‐42 *versus* Aβ1‐42+L200: *P* < 0.001 (**D**), ^#^Aβ1‐42 *versus* Aβ1‐42+L100: *P* < 0.001 and ^##^Aβ1‐42 *versus* Aβ1‐42+L200: *P* < 0.001 (**E**) and ^#^Aβ1‐42 *versus* Aβ1‐42+L100: *P* < 0.01 and ^##^Aβ1‐42 *versus* Aβ1‐42+L200: *P* < 0.0001 (**F**).

Estimated significant overall differences between groups [*F*(3, 16) = 6.89, *P* < 0.01] regarding the AChE‐specific activity in the rat hippocampal homogenates were demonstrated (Fig. 4A). Rats were given Aβ(1‐42) exhibited a significant increase in the AChE activity (*P* < 0.01) as compared to control group. Upon the methanolic extract pre‐treatment, the AChE activity significantly decreased in a dose‐dependent manner as compared to Aβ(1‐42) group.

### Effect of the methanolic extract from *L. capensis* leaves on the SOD and GPX activities

Significant global changes between groups [*F*(3, 16) = 13.57, *P* < 0.001] for the activities of SOD (Fig. 4B) and GPX [*F*(3, 16) = 26.51, *P* < 0.0001; Fig. 4C] in the rat hippocampal homogenates were noticed. Our results demonstrated that rats treated with Aβ(1‐42) showed lower activity for SOD (*P* < 0.01) and for GPX (*P* < 0.01) activities as compared to control group. Moreover, the methanolic extract‐treated groups restored the antioxidant status over the normal conditions observed for the control group.

### Effect of the methanolic extract from *L. capensis* leaves on the total content of reduced GSH, protein carbonyl and MDA levels

Estimated significant overall differences between groups for the total content of reduced GSH (Fig. 4D) with [*F*(3, 16) = 62.20, *P* < 0.0001], for the levels of protein carbonyl (Fig. 4E) with [*F*(3, 16) = 19.57, *P* < 0.0001] and MDA (Fig. 4F) with [*F*(3, 16) = 14.27, *P* < 0.001] in the rat hippocampal homogenates, were demonstrated. In addition, in the Aβ(1‐42) group, low content of the reduced GSH (*P* < 0.0001) along with high levels of protein carbonyl (*P* < 0.0001) and MDA (*P* < 0.01) was demonstrated as compared to the control group as a consequence of impaired antioxidant system. Pre‐treatment of Aβ(1‐42) groups with the methanolic extract significantly restored all these parameters relative to Aβ(1‐42) group.

### Effect of the methanolic extract from *L. capensis* leaves on DNA fragmentation (apoptosis)

**Figure 5:**
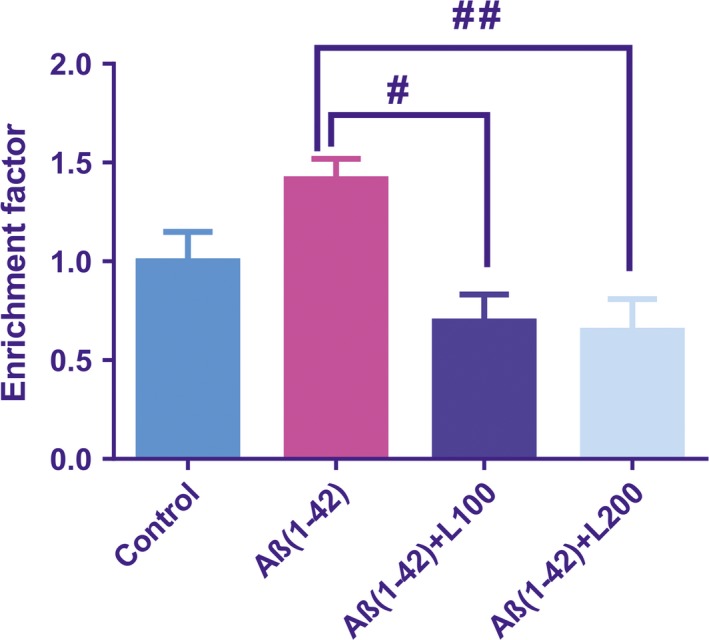
Enrichment factor of apoptosis levels in the Aβ1‐42 groups pre‐treated with the methanolic extract from *Lactuca capensis* leaves (100 and 200 mg/kg). Values are means ± S.E.M. (*n* = 5 animals per group). For Tukey's *post hoc* analyses—^#^Aβ1‐42 *versus* Aβ1‐42+L100: *P* < 0.01 and ^##^Aβ1‐42 *versus* Aβ1‐42+L200: *P* < 0.01

Significant global differences between groups [*F*(3, 16) = 6.43, *P* < 0.01] for DNA fragmentation as demonstrated by enrichment factor (Fig. 5) were demonstrated. The determination of the DNA fragmentation as an important indicator of apoptosis demonstrated a significant increase (*P* < 0.01) of the enrichment factor in the Aβ(1‐42) group as compared to control group. Additionally, administration of the methanolic extract to Aβ(1‐42)‐treated group significantly reduced this factor compared to Aβ(1‐42) group.

### Effect of the methanolic extract from *L. capensis* leaves on BDNF mRNA and IL‐1β mRNA copy number

**Figure 6:**
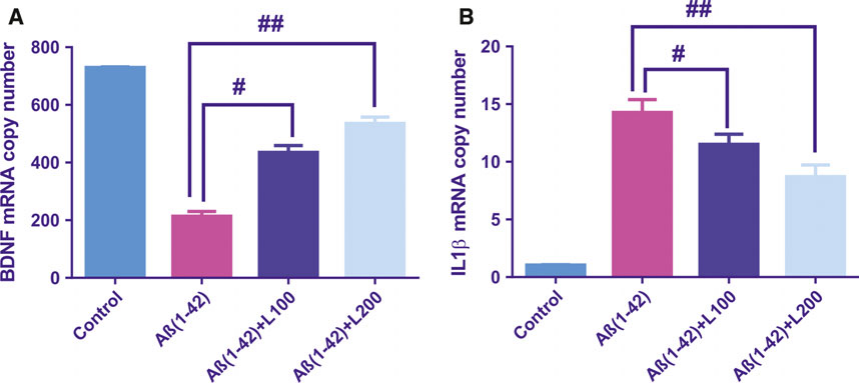
BDNF mRNA copy number (**A**) and IL‐1β mRNA copy number (**B**) in the Aβ1‐42 groups pre‐treated with the methanolic extract from *Lactuca capensis* leaves (100 and 200 mg/kg). Values are means ± S.E.M. (*n* = 5 animals per group). For Tukey's *post hoc* analyses—^#^Aβ1‐42 *versus* Aβ1‐42+L100: *P* < 0.001 and ^##^Aβ1‐42 *versus* Aβ1‐42+L200: *P* < 0.0001 (**A**) and ^#^Aβ1‐42 *versus* Aβ1‐42+L100: *P* < 0.01 and ^##^Aβ1‐42 *versus* Aβ1‐42+L200: *P* < 0.01 (**B**).

Important overall differences between groups for BDNF mRNA copy number (Fig. 6A) with [*F*(3, 16) = 13.52, *P* < 0.0001] and for IL‐1β mRNA copy number (Fig. 6B) with [*F*(3, 16) = 42.08, *P* < 0.001] were demonstrated. We observed an important decreased in BDNF mRNA copy number (*P* < 0.0001) and increased IL‐1β mRNA copy number (*P* < 0.001) in the Aβ(1‐42) group versus the control group. These parameters were significantly reversed by pre‐treatment of Aβ(1‐42) groups versus Aβ(1‐42) group.

A justified correlation between the spontaneous alternation percentage *versus* MDA (*n* = 20, r = −0.752, *P* < 0.001), working memory errors *versus* MDA (*n* = 20, r = 0.853, *P* < 0.01), reference memory errors *versus* MDA (*n* = 20, r = 0.757, *P* < 0.01), SOD *versus* MDA (*n* = 20, r = −0.729, *P* < 0.01), GPX *versus* MDA (*n* = 20, r = −0.866, *P* < 0.001), was demonstrated by linear regression. Moreover, several correlations between the spontaneous alternation percentage *versus* BDNF mRNA copy number (*n* = 20, r = 0.881, *P* < 0.001), working memory errors *versus* BDNF mRNA copy number (*n* = 20, r = −0.808, *P* < 0.01) and reference memory errors *versus* BDNF mRNA copy number (*n* = 20, r = −0.822, *P* < 0.01) were demonstrated by linear regression.

## Discussion

The neuroprotective role and possible action mechanism of the methanolic extract from *L. capensis* leaves in reducing Aβ1‐42‐induced memory deficits, oxidative stress, neuroinflammation and DNA fragmentation, parameters related to AD condition and neurodegeneration were investigated.

Aggregation of the Aβ causes synaptic dysfunction and neurodegeneration especially in the hippocampus, a nervous area involved in memory formation 41. It has been documented that Aβ induced disturbing effects on the overall rat hippocampal neurons and working memory 42, oxidative stress in mouse hippocampus 43 and neuroinflammation 44. Accordingly, to previous data, our results are showing that the rats with a model of Aβ1‐42‐impaired memory significantly decreased their scores during training sessions within Y‐maze and radial arm maze tests 9, 45. Administration of the methanolic extract in both doses, prevented Aβ1‐42‐induced cognitive alteration as demonstrated by increased spontaneous behaviour in Y‐maze test as well as decreased of working and reference memory errors by performing radial arm maze test. These pharmacological responses in behavioural tasks suggest that the methanolic extract could act as a cognitive enhancer agent. Aβ1‐42 treatment decreased both locomotor activity and short‐term memory as previously reported by our group 9. Also, Fedotova *et al*. 46 reported a significant decrease in the locomotor activity in the open‐field test following administration of Aβ25‐35 in intact rats. The methanolic extract effect observed in short‐term memory of Aβ1‐42‐treated rats cannot be attributed to decreased motor activity in Y‐maze test because the percentage of spontaneous alternation was improved and this effect was noticed within radial arm maze test on decreasing of working memory errors. Also, these results were further supported by the decrease of the AChE activity in pre‐treated Aβ1‐42 rats with the methanolic extract, suggesting that the plant extract could act as an unspecific enhancer of the cholinergic activity. By contrast, increased AChE activity was demonstrated in the Aβ1‐42 rats versus the control group. Studies suggested that AChE promotes beta‐amyloid aggregating plaques in the cerebral cortex of the patients with AD 47. *In vitro* studies have been shown that AChE stimulates the deposition of insoluble fibrils containing both Aβ and AChE, which exhibited a higher toxicity to the cells rather than Aβ alone 48. Therefore, AChE is the target of the cholinesterase inhibitors used for addressing the cholinergic deficits in AD 49.

HPLC analyses of the extract indicated the presence several flavonoids and hydroxycinnamic acids. Among them, the most important components isolated were mainly catechin (2.4958 mg/g of dry extract), rutoside (2.5915 mg/g of dry extract), caffeic acid (1.7579 mg/g of dry extract) and rosmarinic acid (5.2784 mg/g of dry extract). Catechin, a flavonoid, exerts anti‐AChE activity 50 and protective effects on the hippocampal formation and spatial memory in ageing rats 51. Rutoside, a phenolic compound, has been reported to inhibit cholinergic and monoaminergic enzymes with relevance for the progress of neurodegenerative disease 52. The polyphenolic compound, caffeic acid, was previously reported to possess strong antioxidant and anti‐AChE activities and attenuated memory deficits in intracerebroventricular streptozotocin‐induced experimental dementia in rats 53. Finally, rosmarinic acid, a polyphenol similar to caffeic acid, improved amyloid‐β25‐35‐induced cognitive impairment in mice 54 and exhibited anti‐AChE activity in rats 55. In the light of these results, the cognitive‐enhancing and anti‐AChE potential of the methanolic extract are mostly attributed to the presence of the aforementioned compounds.

We assessed the antioxidant effect of the methanolic extract in the rat hippocampus homogenates as oxidative stress contributes to pathogenesis and histological changes in patients with neurodegenerative afflictions 56. Evidence reported a link between increasing of oxidative stress and AD due in part to the enhanced production of oxygen species and loss of activity of various antioxidant enzymes 57, 58, 59. Endogenous enzymes with known antioxidant activities such as SOD and GPX are firstly involved against cellular oxidative damage 60. These antioxidants exhibit a critical role in scavenging reactive oxygen species, reduction in hydrogen peroxide and maintaining redox balances in the biological system. In our study, the enzymatic antioxidants SOD and GPX have significantly decreased in Aβ1‐42 administered rats which were restored to the normal conditions following pre‐treatment of Aβ1‐42 with the methanolic extract. Furthermore, the total content of reduced GSH has much lowered in Aβ1‐42 administered rats versus control rats. However, administration of the methanolic extract in Aβ1‐42 rats improved the GSH reduced content near the normal level. GSH act as an antioxidant agent playing an essential role in protecting the cells against lipid peroxidation 61. Lipid peroxidation is involved in the pathogenesis of Alzheimer's disease and other selected age‐related neurodegenerative disorders 62. Oxidative protein damage was shown to be implicated in the pathogenesis of dementia disorders such as AD 63. In this study, the rat hippocampal homogenates showed significantly increased MDA (lipid peroxidation) and protein carbonyl (protein oxidation) levels from Aβ1‐42 rats as compared to control groups. Pre‐treatment of Aβ1‐42 with the methanolic extract significantly lowered MDA and protein carbonyl levels close to normal levels. Our results demonstrated that the methanolic extract has an antioxidant potential by contributing to improving the memory function in AD rat models.

It has been documented that ageing is an important risk factor for AD. When neurons age, they become sensitive to cell damage induced by Aβ42 oligomers that promote apoptosis 64. In our study, pre‐treatment of Aβ1‐42 with the methanolic extract was capable of attenuating hippocampal apoptosis, as demonstrated by a lower enrichment factor relative to Aβ1‐42‐treated rats that were consistent with its anti‐apoptotic activity in two neuron‐like cells, such as N2a and PC12 65.

To evaluate the role of the methanolic extract in the neurogenesis and inflammation, we examined BDNF mRNA and IL‐1β mRNA copy numbers in the rat hippocampus. An important role of the BDNF in synaptic plasticity and neuronal survival was shown in the literature 66. Supporting evidence reveals that there is a link between decreased BDNF expression in human brains and AD pathogenesis. Additionally, several studies showed reduced mRNA and BDNF protein levels in the serum and brain of patients with AD relative to healthy aged controls 66, 67, 68. Moreover, the experiments performed *in vivo* demonstrated that BDNF protein has a neuroprotective role against the cytotoxic effects and learning impairments induced by Aβ1‐42 in rats 69. Also, in a TgCRND8 transgenic AD mouse model, increased levels of Aβ1‐42 are related with decreased BDNF levels 70. According to with these data from the literature, our results revealed decreased BDNF mRNA copy number in Aβ1‐42‐treated rats as compared to control group. As expected, pre‐treatment of Aβ1‐42 rats with the methanolic extract reversed the BDNF mRNA copy number close to normal conditions. Previous studies have demonstrated that Aβ has a coreleasing role for a macrophage proinflammatory cytokine, IL‐1β, providing support that the chronic inflammation is an ongoing pathological process encompassing senile plaques in AD 71. *In vitro*, IL‐1β is realized by activated microglia after stimulation with Aβ 72. Moreover, IL‐1β modulated APP expression and proteolysis 73. In our study, increased IL‐1β mRNA copy number by IL‐1β in the rat hippocampal tissue was significantly decreased by the methanolic extract pre‐treatment.

Finally, when linear regression was determined, a correlation between the spontaneous alternation percentage *versus* MDA, working memory errors *versus* MDA, reference memory errors *versus* MDA, SOD *versus* MDA, GPX *versus* MDA, the spontaneous alternation percentage *versus* BDNF mRNA copy number, working memory errors *versus* BDNF mRNA copy number and reference memory errors *versus* BDNF mRNA copy number was demonstrated. Our results suggested that improving behavioural scores in specific tests are related to decreasing of oxidative damage together with increasing of BDNF mRNA copy number in the hippocampal tissue of pre‐treated Aβ1‐42 rats with the methanolic extract.

Regarding the limitation of our study, it remains unknown whether a single component of the methanolic extract mediated the observed effects or whether the effects are due to an interaction of all components. In addition, our study provides further support for studying the methanolic extract action at the cellular and molecular level.

## Conflict of interest

The authors confirm that there are no conflict of interests.
